# Zinc Deficiency Disrupts Germ Cell Nest Breakdown During In Vitro Ovary Culture

**DOI:** 10.1002/mrd.70092

**Published:** 2026-02-12

**Authors:** James M. Hester, Suzanne M. Getman, Melissa E. Pepling, Francisco J. Diaz

**Affiliations:** ^1^ Integrative and Biomedical Physiology Program The Pennsylvania State University University Park Pennsylvania USA; ^2^ Department of Animal Science The Pennsylvania State University University Park Pennsylvania USA; ^3^ Department Biology Syracuse University Syracuse New York USA

**Keywords:** follicle, ovary, zinc

## Abstract

In mammals, the size of the non‐renewable primordial follicle pool is established before or soon after birth. Primordial follicles, each composed of a single oocyte surrounded by somatic cells, are the only source of gametes during the entire reproductive lifespan of the female. The size of the initial follicle pool is a key component in determining reproductive longevity, and impaired follicle assembly has severe consequences for fertility. Here, we evaluate the effect of zinc deficiency on germ cell survival and follicle activation in late pregnancy and early postnatally using in vitro organ culture of mouse ovaries. Zinc deficiency did not affect apoptosis, germ cell number, and gene expression in fetal ovaries. Germ cell nest breakdown, which occurs in the newborn mouse ovary, involves invasion of germ cell cysts by somatic cells to form primordial follicles was disrupted by zinc deficiency leading to fewer oocytes enclosed in follicles (*p* = 0.002). Gene expression of both an oocyte‐specific factor (*Bmp15*) and granulosa cell–specific factor (*Foxl2*) shown to regulate nest breakdown was decreased by zinc deficiency (*p* < 0.05). There was also a strong trend toward fewer activated growing follicles after zinc‐deficient culture of newborn ovaries (*p* = 0.051). The initial wave of follicle activation is key for both paracrine and endocrine signaling from the ovary. Disruption of this process, as well as impaired primordial follicle formation, may impact subsequent fertility.

## 1 Introduction

In mammals, female germ cells divide mitotically only during fetal development. Therefore, at birth, the ovary contains the lifetime supply of oocytes available for reproduction (Pepling 2006). The oocytes are surrounded by somatic pregranulosa cells to form structures known as primordial follicles—a dormant structure that may persist for months in mice or decades in humans. The number of follicles in the ovary at any time is referred to as the ovarian reserve of quiescent primordial follicles. This contrasts with the meaning of the ovarian reserve in a clinical setting that reflects the abundance of growing follicles, which indirectly reflects how many primordial follicles are present. The initial size and maintenance of the primordial follicle pool is the determining factor in reproductive longevity (Depmann et al. 2015), as the depletion of the ovarian reserve leads to reproductive senescence (Tingen et al. 2009). Factors that affect primordial follicle assembly and activation are therefore intriguing targets for understanding and treating infertility. In particular, this line of research holds promise for treating Primary Ovarian Insufficiency (POI), a disorder in 1% of women characterized by rapid depletion of the ovarian reserve leading to early menopause onset (Coulam et al. 1987; De Vos et al. 2010). Prenatal and neonatal nutrition have been shown to affect the formation of the follicle reserve, as starvation in rodent models reduces primordial follicle numbers while increasing germ cell apoptosis (Y.‐Y. Wang et al. 2017). The role of specific micronutrients during this period is less clear.

The gestation period of the mouse is 20–22 days. Primordial germ cells first appear on embryonic Day 8 (E8), reach the developing gonad on E10, and divide mitotically until E13.5 (Monk and McLaren 1981). At the end of mitosis, the cells are found in germ cell cysts formed from incomplete cytokinesis during division (Pepling and Spradling 1998). Beginning on E13.5, the germ cells enter meiosis under the influence of retinoic acid arising from the mesonephros (Koubova et al. 2006). Germ cells pass through the early stages of meiosis, including recombination, before arresting at the diplotene stage of meiotic prophase I. The first diplotene oocytes appear on E17.5, and the % of diplotene oocytes increases over the next 4 days until all oocytes reach diplotene arrest (Dutta et al. 2016). In mid to late gestation, depending on the species, germ cell nests begin to break down and form primordial follicles composed of one oocyte and a surrounding complement of somatic cells (Pepling et al. 2010). The loss of estrogen signaling is the likely initiator of nest breakdown in rodents (Kezele and Skinner 2003; Dutta et al. 2014). A disruption of either meiotic progression or germ cell nest breakdown may compromise fertility. After assembly, the majority of primordial follicles become dormant until paracrine signals activate the follicle to enter a growth phase (McLaughlin and McIver 2009). However, a subset of medullary follicles activate immediately after assembly with no dormant phase (Zheng, Zhang, Gorre, et al. 2014). These follicles are unlikely to ovulate but produce both endocrine and paracrine factors that are hypothesized to promote development of the hypothalamic–pituitary–ovarian signaling axis, as well as regulate the activation in the dormant follicle pool (Zheng, Zhang, and Liu 2014).

Increasing evidence points to zinc homeostasis as an important pathway regulating follicular development. Zinc plays an integral role in oocyte development, particularly during preantral follicle growth (Chen et al. 2023; Hester and Diaz 2025) and during ovulation and fertilization (A. M. Kim et al. 2011; Tian and Diaz 2012, 2013; Tian et al. 2014). In the fully developed ovulatory follicle, proper zinc regulation is necessary to control meiotic division (A. M. Kim et al. 2010; Bernhardt et al. 2011, 2012; Kong et al. 2012; Zhao et al. 2014), but its role in the early stages of ovarian development has not been examined in mammals. A zinc requirement for oocyte and follicle development in the fetal ovary would be a significant finding, as 82% of pregnant women worldwide consume less than the recommended dietary allowance for zinc (Caulfield et al. 1998).

**Figure 1:**
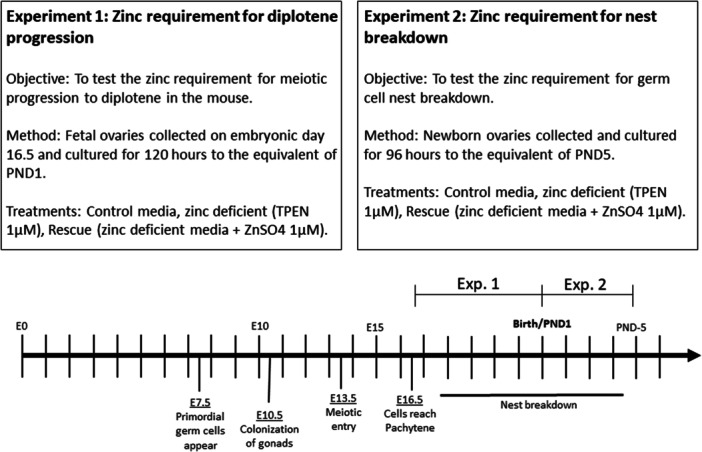
Study outline.

To evaluate the effects of zinc deficiency on germ cell survival and nest breakdown, we have employed an in vitro organ culture system using mouse ovaries. Zinc‐deficient media were created with the addition of the zinc‐specific chelator TPEN (*N*,*N*,*N*′,*N*′‐tetrakis[2‐pyridinylmethyl]‐1,2‐ethanediamine). We conducted two separate studies using either fetal or newborn mouse ovaries (Figure 1). Establishing a zinc requirement for the fetal ovary will provide new insight into female fertility and may have implications for nutritional recommendations and micronutrient supplementation during pregnancy.

## 2 Methods

### 2.1 Animals

CD‐1 mice (*Mus musculus*) were commercially obtained from Charles River Laboratory (Wilmington, MA). Animal care and handling were reviewed and approved by the Institutional Animal Care and Use Committee of Penn State. Animals were maintained on a 14:10 light/dark cycle and fed ad libitum. One adult male and one adult female were housed per cage. Females were checked for the presence of a vaginal mating plug each morning to monitor pregnancy onset. The morning of plug discovery was designated as E0.5. To collect fetal ovaries, pregnant dams were euthanized by CO_2_ asphyxiation followed by cervical dislocation on E16.5. Parturition most often occurred on the equivalent of E21.5 and was designated postnatal Day 1 (PND1). To collect newborn ovaries, pups were euthanized by decapitation on PND1 before dissection of the ovaries.

### 2.2 Ovary Culture

Ovaries were collected from fetal and newborn mice immediately after euthanasia and stored in Hank's Balanced Salt Solution on ice until collection was complete. Ovaries were cultured on a porous polycarbonate membrane floated on culture media. Culture media was distinct for fetal and newborn ovaries. For fetal ovary culture, ovaries were cultured in DMEM–Ham's F‐12 media supplemented with penicillin–streptomycin, ITS, 0.1% BSA, 0.1% albumax, 0.05 mg/mL l‐ascorbic acid, 10 µM estradiol, and 10 µM progesterone (hormones added to prevent premature nest breakdown) as described (Pepling et al. 2010). DMEM/F‐12 has a reported concentration of 1.5 μM zinc (Gibco #11320033). Newborn ovaries were cultured as described (O'Brien et al. 2003) in Waymouth 752/1 medium supplemented with 0.23 mM pyruvic acid, penicillin–streptomycin, and 10% fetal bovine serum. Although Waymouth medium has no added zinc, we detected 0.64 μM zinc by ICP‐MS (Measured at the Laboratory for Isotopes and Metals in the Environment [LIME] at the Pennsylvania State University). Zinc‐deficient media were created by adding the zinc‐specific intracellular chelator TPEN (Sigma) dissolved in DMSO to a concentration of 1 µM. The concentration of zinc was chosen because 10 μM but not 1 μM TPEN induced cell death in ovarian cells after 24 h (Chen et al. 2023). Rescue media contained 1 µM TPEN and 1 µM ZnSO_4_ (sigma). Fetal ovaries were cultured for 5 days to the equivalent of E21.5. Newborn ovaries were cultured for 4 days to the equivalent of PND5.

### 2.3 Histology

After organ culture, ovaries were fixed in Bouin's fixative for 2 h at room temperature, followed by an ethanol dehydration series. Ovaries were paraffin‐embedded with the assistance of the Penn State microscopy and histology core facility. Paraffin blocks were serially sectioned at 4 µm and stained with hematoxylin and eosin according to standard procedures. Stained sections were imaged with a Nikon Eclipse TE200 inverted microscope fitted with an Olympus DP20 brightfield camera. Every fifth section was analyzed as follows: For cultured newborn ovaries, every fifth section was analyzed by three independent researchers, two of whom were blinded to treatment. Total oocyte number, oocytes in nests (two or more oocytes proximal to each other with no intervening somatic cells), primordial follicles (one oocyte completely surrounded by granulosa cells), and activated follicles (identified by cuboidal granulosa cells surrounding the oocyte) were counted per section. An average of seven sections were analyzed per ovary. Data from all three researchers were averaged for each tissue section. Section counts were combined to provide counts per ovary. Five ovaries were analyzed per treatment group. To calculate oocyte density (oocytes/area), the surface area of each tissue section was calculated with ImageJ. Cultured fetal ovaries were fixed, embedded, sectioned, and stained identically to newborn ovaries. For fetal ovary analysis, total oocyte count, total diplotene oocytes, and total prediplotene oocytes were counted on every fifth tissue section. The researcher was blinded to the treatment group before analysis. An average of 9 sections were analyzed per fetal ovary, and 2–5 ovaries were evaluated per treatment. To eliminate the possibility of double‐counting oocytes, only cells displaying nuclear staining were quantified in either the newborn or fetal ovaries.

### 2.4 Total RNA Extraction and qPCR

After culture, ovaries were snap frozen and stored at −80°C before RNA extraction. Total RNA was extracted with the Absolutely RNA Microprep kit (Agilent Technologies) and reverse transcribed into cDNA using quantitect RT kit (Qiagen). cDNA was evaluated for stable expression of three housekeeping genes—*Rpl19*, *Gapdh*, and *βactin*—by qPCR. Only cDNA samples that demonstrated a consistent housekeeping gene expression pattern were used for further analysis. Gene expression was calculated for 3–4 ovaries per treatment according to the ddct method (Livak and Schmittgen 2001) normalized to the geometric mean of Ct values from the three housekeeping genes (Vandesompele et al. 2002). Primer sequences are listed in Table 1. Amplification products were sequenced to validate primer specificity.

**Table 1:** qPCR primers.

Gene symbol	Forward primer (5′–3′)	Reverse primer (5′–3′)
*Scp‐1*	GTCAAGTGTCCGCCGTGAAA	CCTGATAGTGACAACTGCCAGA
*Scp‐3*	TCGGGGCCGGACTGTATT	AAGGTGGCTTCCCAGATTTCC
*Msy2*	AAGTCCTGGGCACAGTCAAAT	CTCCCCATCTCCAACACTCC
*Spo11*	AGCATGAAGTGTCTCACTAGCA	CATTAACAGGGCAAGGCACCTA
*Dmc1*	CCCTCTGTGTGACAGCTCAAC	GGTCAGCAATGTCCCGAAG
*Ddx4*	GAAGAAATCCAGAGGTTGGC	GAAGGATCGTCTGCTGAACA
*Dazl*	GATGGACATGAGATCATTGGAC	ATACCAGGGAGCAATCCTGAC
*Bcl2*	GAACTGGGGGAGGATTGTGG	GCATGCTGGGGCCATATAGT
*Bax*	GATCCAAGACCAGGGTGGCTG	TCCCCTTCCCCCATTCATCC
*Mcl‐1*	TGTAAGGACGAAACGGGACT	AAAGCCAGCAGCACATTTCT
*Atg7*	CCTGCACAACACCAACACAC	CACCTGACTTTATGGCTTCCC
*Bmp15*	ACACAGTAAGGCCTCCCAGA	GATGAAGTTGATGGCGGTAAA
*Gdf9*	CTACAATACCGTCCGGCTCT	CAAGTGTTCCATGGCAGTCA
*Activin*	GGGTAAAGTGGGGGAGAACG	ACTTCTGCACGCTCCACTAC
*Amh*	AGCTGGACACCATGCCTTT	TTCGAAGCCTGGGTCAGA
*Amhr2*	TTACAGCCATCTGCCTCCTT	TCAGCAACAACACGAGAAACA
*Nobox*	AGGGACGTTCCTGGCAGT	GCTGCTTGCTTGGTAGTCCT
*Figla*	ACAGAGCAGGAAGCCCGTA	GTCAGAGGGTCTGCCACTGT
*Foxl2*	AACACCGGAGAAACCAGACC	CGTAGAACGGGAACTTGGCT
*Kitl*	CAAGGAGATCTGCGGGAAT	CAATGACTAGGCAAAACATCCA
*Pten*	TCTGCCATCTCTCTCCTCCTT	TTCTGCAGGAAATCCCATAGCAA
*Notch2*	AGCAGGAGCAGGAGGTGATA	TGGGCGTTTCTTGGACTCTC
*Wnt4*	CCTGCGACTCCTCGTCTT	TCTGGATCAGGCCTTTGAGT

### 2.5 GCNA and Cleaved PARP Immunohistochemistry

Ovaries were fixed in 5% electron microscopy‐grade paraformaldehyde (Electron Microscopy Sciences) overnight at 4°C and stained as previously described (Pepling and Spradling 1998; T. Sun et al. 2015). Whole ovaries were incubated with GCNA antibody (TRA98, BBridge) diluted 1:100 to label germ cells (Tanaka et al. 1998; Grive et al. 2014) and with cleaved PARP (product no. E51; Abcam) diluted 1:100 to label cells undergoing apoptosis overnight, and then with goat anti‐rat secondary Alexa 488 and goat anti‐rabbit Alexa 568, respectively. Nuclei were labeled with TOTO‐3 (Invitrogen). A confocal microscope (model LSM 710; Zeiss) was used to image the ovaries. Each ovary was assigned a randomly selected core for visualization. A core consists of four optical sections that are 212 × 212 µm and are each separated by a depth of 15–20 µm. The four optical sections span both the cortex and medulla to ensure that all regions of the ovary are assessed. Zen software (Zeiss) was used for imaging and subsequent analysis of the ovaries.

### 2.6 Germ Cell Counting

Total oocytes (GCNA‐positive) and apoptotic oocytes (cleaved PARP‐positive) were determined by counting the number of cells found within each of four optical sections per ovary. Germ cell counts were combined from each section to give the oocyte count per section core. Data from five ovaries were averaged per treatment. Since data from a limited number of sections were used as a proxy for the total oocytes per ovary, only ovaries that appeared to be of similar size and depth were used for this analysis.

### 2.7 Statistics

The treatment effect was analyzed by one‐way ANOVA for all experiments. For significant results, differences between groups were evaluated using Tukey's LSD method. All results are presented as mean ± SEM.

## 3 Results

### 3.1 Zinc Deficiency Does Not Alter Oocyte Number or Rate of Apoptosis During Fetal Ovary Culture

**Figure 2:**
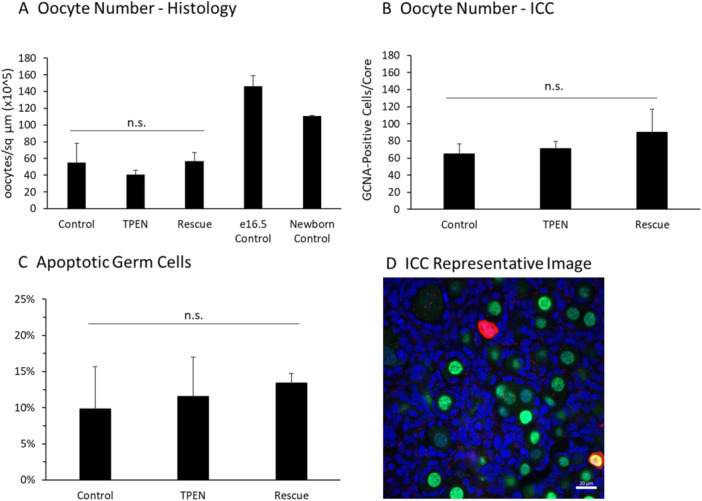
TPEN does not alter oocyte number or apoptosis in the fetal ovary. (A) Oocyte number in fetal ovaries cultured in control media, TPEN, or zinc rescue treatment groups and stained with H&E for oocyte counting (*p* = 0.443; *n* = 9 histological sections per fetal ovary). E16.5 and newborn ovary controls are shown for reference but were not included in the analysis. (B) Germ cell number per optical core in whole ovaries from control, TPEN, and rescue treatment groups stained with GCNA antibody (*p* = 0.537). (C) Proportion of apoptotic germ cells in whole ovaries were labeled with an antibody to cleaved PARP1 (*p* = 0.887). (D) Representative image of whole mount ovary stained with GCNA and cleaved PARP1. Green: GCNA; red: cleaved PARP1; blue: TOTO‐3 nuclear stain. Scale bar = 20 mm.

Total oocyte number was quantified in two ways. Oocytes were counted during histological analysis of fetal ovaries, and whole ovaries were labeled with an antibody to the germ cell marker GCNA, followed by confocal optical sectioning. Both experimental methods showed that treatment did not affect the number of germ cells in the ovary after 5 days of culture. For histological analysis, oocytes were calculated per sq micron. The result from each treatment was multiplied by a factor of 10^5^ to simplify presentation (Control: 54.8 ± 23.1; TPEN: 40.5 ± 5.7; Rescue: 57.0 ± 10.1 ooc/sq μm × 10^5^; *p* = 0.443; Figure 2A). For whole ovary immunostaining, GCNA‐positive germ cell counts were combined from 4 sections per ovary and averaged for 4–5 ovaries per treatment (Control: 65.0 ± 11.5; TPEN: 71.2 ± 8.3; Rescue: 90.25 ± 26.6 germ cells/ovary; *p* = 0.537; Figure 2B). Apoptotic cells were labeled in fetal ovaries after 5 days of culture using an antibody to cleaved PARP1. As expected from the cell counting data, the rate of apoptosis was not different between treatments (Control: 9.8% ± 5.9%; TPEN, 11.6% ± 5.4%; Rescue, 13.4% ± 1.2%; *p* = 0.887; Figure 2C).

### 3.2 Zinc Deficiency Does Not Alter Gene Expression in the Fetal Ovary

**Figure 3:**
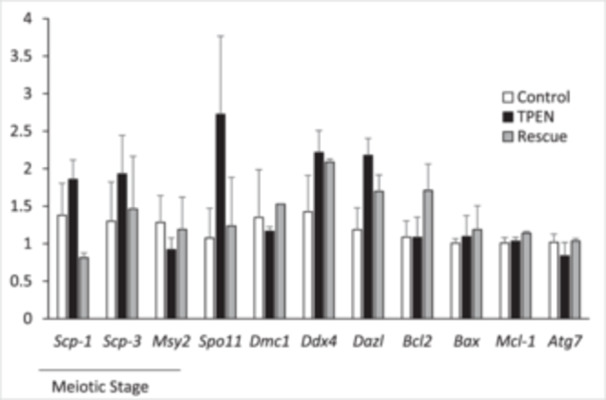
Zinc status does not affect gene expression in the fetal ovary. Gene expression of stage‐specific meiotic markers, meiotic recombination genes, and cell death regulators in control, TPEN, and rescue treatment groups was evaluated by qPCR (fold change from control). Three to four ovaries were analyzed per treatment.

Gene expression was compared between groups using qPCR (Figure 3). Zinc status did not affect gene expression of meiotic stage markers (*Scp1*, *Scp3*, and *Msy2*), genes governing meiotic recombination (*Spo11*, *Dmc1*, *Ddx4*, and *Dazl*), or genes governing apoptosis/autophagy (*Bcl2*, *Bax*, *Mcl1*, and *Atg7*). Expression was measured as the fold change from control (*p* > 0.05 for all genes).

### 3.3 Zinc Deficiency Does Not Alter Oocyte Number in Cultured Newborn Ovaries

**Figure 4:**
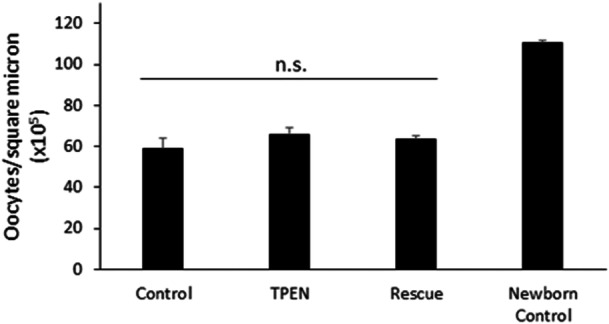
TPEN does not alter oocyte number after newborn ovary culture. Oocyte number expressed as oocyte/square micron (×10^5^) in newborn ovaries cultured for 4 days in control, TPEN, and rescue media (*p* = 0.429; *n* = 7 histological sections per ovary, five ovaries per group).

Oocyte number was evaluated with histology after 4 days of newborn ovary culture in treated media. Oocyte number/area was evaluated according to the same procedures detailed for fetal ovary histology. Treatment did not affect oocyte number (Control: 58.7 ± 5.3; TPEN: 65.9 ± 3.4; Rescue: 63.1 ± 1.8 ooc/sq μm × 10^5^; *p* = 0.429; Figure 4). All cultured oocytes contained fewer oocytes than freshly collected newborn ovaries (in vivo control) (110.5 ± 0.9 ooc/sq μm × 10^5^).

### 3.4 Zinc Deficiency Impairs Nest Breakdown During Newborn Ovary Culture

**Figure 5:**
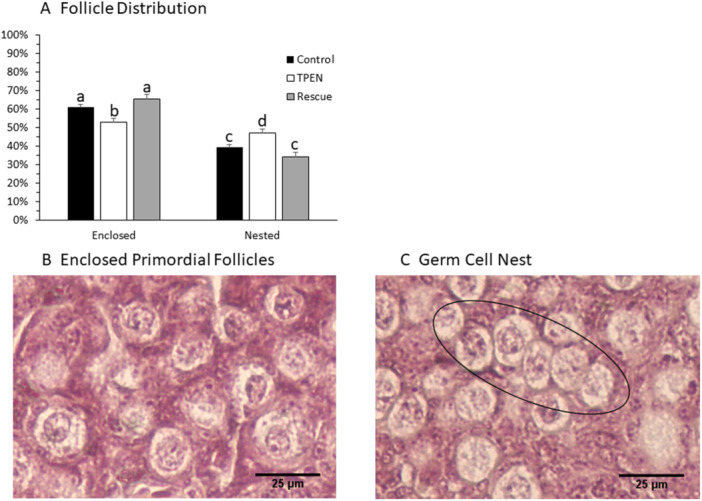
TPEN impairs germ cell nest breakdown in newborn ovary culture. (A) Follicle distribution in control, TPEN, and rescue ovaries after 4 days in culture (*p* = 0.002; *n* = 7 histological sections per ovary, five ovaries per group). (B) Representative image of fully enclosed primordial follicles. (C) Representative image of a germ cell nest (oval).

After 5 days of culture, newborn ovaries were processed for histology and stained with hematoxylin and eosin. Ovaries cultured in TPEN‐treated media contained fewer enclosed primordial follicles and a higher proportion of oocytes in germ cell nests. (Figure 5; percent of nested oocytes—Control: 9.2 ± 1.6; TPEN: 47.1 ± 2.1; Rescue: 34.3 ± 2.2; *p* = 0.002).

### 3.5 Zinc Deficiency May Reduce the Size of the Initial Follicular Wave

**Figure 6:**
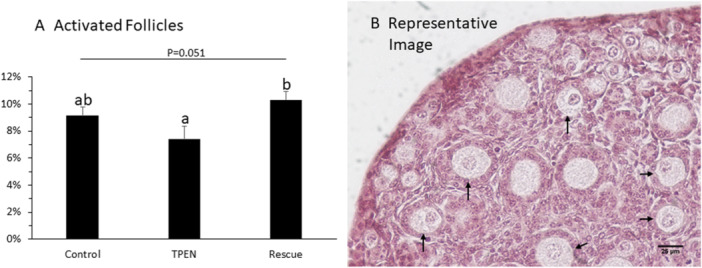
TPEN may reduce the size of the initial follicle wave. (A) Proportion of activated follicles in control, TPEN, and rescue groups (*p* = 0.051; *n* = 7 histological sections per ovary, five ovaries per group). Pairwise comparisons show a statistical difference between TPEN and Rescue treatment, indicating a possible zinc‐mediated effect. (B) Representative image of ovary with activated follicles containing visible oocyte nuclei (arrows).

The number of activated follicles (identified by association with cuboidal granulosa cells) was counted in newborn ovary sections after H&E staining (Figure 6). There was a strong trend (*p* = 0.051) toward a treatment effect on the proportion (%) of activated follicle. Tukey pairwise comparisons were used to test differences between treatment groups. TPEN and Rescue treated ovaries displayed a significantly different rate of activation (TPEN: 7.4% ± 1.0%; Rescue: 10.3 ± 0.6%; *p* < 0.05). The activation rate in control ovaries (9.2% ± 0.6%) was not significantly different from either TPEN or Rescue.

### 3.6 TPEN Treatment Reduces Expression of *Bmp15* and *Foxl2* in Cultured Newborn Ovaries

**Figure 7:**
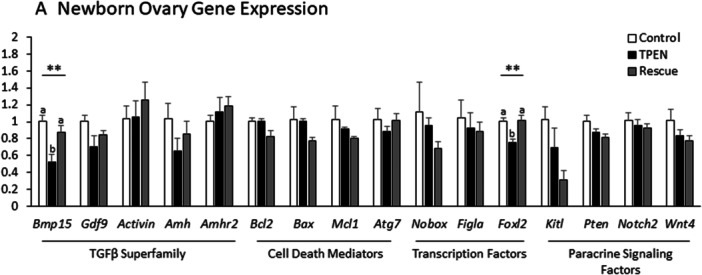
TPEN reduces expression of *Bmp15cl2* and *Foxl2* in cultured newborn ovaries. (A) Abundance of transcripts previously shown to regulate nest breakdown in control, TPEN, and rescue treatment groups was evaluated by qPCR (*p* < 0.01 for *Foxl2* and *Bmp15*). Expression shown as fold change from control. Three to four ovaries were analyzed per treatment.

Gene expression was compared between treatment groups by qPCR according to the ddct method. Zinc deficiency reduced the expression of the oocyte secreted TGFβ family member *Bmp15* and the somatic cell transcription factor *Foxl2* (*p* < 0.01 for each). Other factors previously shown to regulate nest breakdown were also evaluated but showed no change between treatments (Figure 7).

## 4 Discussion

Primordial follicle assembly involves meiotic progression of the oocyte as well as germ cell nest breakdown and invasion of somatic cells. These two processes are closely related (Paredes et al. 2005), but not dependent processes—meaning neither is a prerequisite for the other (Dutta et al. 2016). We tested the effect of zinc deficiency on meiotic progression and germ cell nest breakdown independently as these processes are distinct in the mouse, and both contribute to primordial follicle formation. It is important to note that nest breakdown in humans occurs in utero concurrently with meiotic progression (Bendsen et al. 2006). The results of this study indicate that zinc deficiency impairs germ cell nest breakdown and may impair follicle activation of the initial medullary follicular wave.

TPEN treatment did not affect the rate of germ cell apoptosis during either fetal or newborn ovary culture. This result is surprising given the already high rate of apoptosis during primordial follicle formation. In mice, only one‐third of germ cells will survive to become primordial follicles (Pepling and Spradling 2001), while the survival rate in humans is even lower (Vaskivuo et al. 2001). Oocyte survival appears to be regulated by the Bcl2 family of apoptotic regulators. The balance of proapoptotic (BAX) and antiapoptotic (BCL2, MCL1) factors determines cell fate during primordial follicle assembly (Perez et al. 1999; Greenfeld et al. 2007; Jones and Pepling 2013b) in conjunction with regulation of autophagy (Gawriluk et al. 2011). Zinc impacts apoptosis rates in a variety of cell types, primarily promoting cell survival (Thomas and Caffrey 1991; Perry et al. 1997; W. Sun et al. 2018). Zinc chelation did not affect the apoptosis rate in oocytes. The consistent apoptotic rate across treatments was demonstrated by cell counting in stained histology sections, whole mount ICC, and gene expression assays. These results demonstrate that the apoptotic rate of oocytes is tightly regulated even during environmental perturbations.

TPEN treatment did affect germ cell nest breakdown during newborn ovary culture. TPEN‐treated ovaries contained a higher percentage of oocytes in nests and fewer primordial follicles after 5 days of treatment. Whether the rate of nest breakdown was merely slowed by TPEN treatment or permanently disrupted in a subset of oocytes is unclear from this experiment. Equally remarkable is the effect of zinc deficiency on activation of the initial follicular wave. This unique class of follicles is the first to be assembled and plays a vital role in controlling ovarian function before puberty in mammals (Zheng, Zhang, Gorre, et al. 2014; Zheng, Zhang, and Liu 2014). The initial follicle wave is important in providing endocrine feedback to the pituitary, as well as inhibiting overactivation of dormant primordial follicles via paracrine inhibition. The impact of fewer follicles in the initial follicular wave remains to be investigated. Reduced activation of this class of follicles could be hypothesized to disrupt signaling in the hypothalamic–pituitary–gonadal axis as well as lead to over‐recruitment of dormant primordial follicles, which may lead to premature ovarian insufficiency.

Nest breakdown requires the coordination of multiple signaling pathways among a variety of cell types. To identify a signaling pathways vulnerable to zinc deficiency, we evaluated the effect of zinc deficiency on the expression of 16 genes that have been previously shown to regulate nest breakdown (Vainio et al. 1999; Soyal et al. 2000; Yan et al. 2001; Rajkovic et al. 2004; Uda et al. 2004; J. Wang and Roy 2004; Bristol‐Gould et al. 2006; Greenfeld et al. 2007; Reddy et al. 2008; Trombly et al. 2009; Gawriluk et al. 2011; Nilsson et al. 2011; J. Y. Kim 2012; Jones and Pepling 2013a, 2013b). TPEN treatment downregulated transcription of the oocyte‐derived TGFβ superfamily member *Bmp15* (Bone Morphogenic Protein 15) and the somatic cell transcription factor *Foxl2* (Forkhead Box L2). Oocytes secrete BMP15 along with another TGFβ member GDF9 (Growth Differentiation Factor 9). BMP15 and GDF9 activate SMAD proteins in the surrounding somatic cells (Moore et al. 2003). SMAD activation directs cell differentiation, mitosis, and survival. Previous studies in our lab have shown that zinc deficiency reduces *Gdf9* expression in fully grown oocytes (Tian and Diaz 2013). The reduction of *Bmp15* expression seen here indicates that zinc deficiency impairs TGFβ signaling at multiple developmental stages. BMP15 or GDF9 knockout mice display an increased incidence of follicles containing multiple oocytes, indicating impaired nest breakdown (Yan et al. 2001).

Ovarian morphology is even more disrupted in *Foxl2* knockout mice in which entire germ cell nests remain in the adult ovary (Uda et al. 2004). In addition, follicles activate prematurely in the knockout mice due to a lack of inhibitory signaling from the granulosa cells, leading to rapid follicle loss. In humans, mutations in Foxl2 result in blepharophimosis/ptosis/epicanthus inversus syndrome (BPES), a rare disease characterized by eyelid deformation and premature ovarian insufficiency (Crisponi et al. 2001). Foxl2 activity is key to controlling granulosa cell proliferation, differentiation, and development (Schmidt et al. 2004; Kuo et al. 2012), and contributes to premature ovarian insufficiency in humans.

## 5 Conclusion and Future Directions

This study has demonstrated that zinc deficiency impairs germ cell nest breakdown and follicle assembly in the newborn mouse ovary. Two genes previously shown to be required for nest breakdown were downregulated by TPEN treatment. Oocyte apoptosis, a key factor in primordial follicle assembly, was not affected by zinc status in either the fetal or newborn ovaries. Interestingly, zinc deficiency may reduce the size of the initial follicular wave, which plays an important role in the prepubertal female. The long‐term effects of disrupting the initial follicular wave are unknown.

Future studies should test the effect of in vivo zinc deficiency on primordial follicle assembly. Inducing marginal zinc deficiency during fetal or neonatal development is complicated by homeostatic regulation of zinc status. Zinc absorption is upregulated in the intestine in response to low dietary intake (Hambidge et al. 2010) or increased physiological need such as lactation (Fung et al. 1997). Zinc transfer from the maternal circulation to the placenta is also highly regulated such that the zinc level in serum collected from the umbilical cord is unchanged over a wide range of circulating maternal zinc levels (Vargas Zapata et al. 1997). These homeostatic mechanisms make it difficult to induce marginal zinc deficiency in experimental models. Genetic ablation of zinc transporters in the placenta or fetal ovary may allow researchers to induce fetal zinc deficiency without altering maternal zinc status. Regardless of the method, in vivo zinc deficiency effects on primordial follicle assembly will allow subsequent fertility evaluation in the adult animal. This step is required to test if fetal or neonatal zinc deficiency can impact ovarian status and fertility in the adult.

## Author Contributions

J.M.H. and F.J.D. designed the study. J.M.H. carried out experiments, collected samples, and analyzed data. S.M.G. performed immunostaining assays and imaging. J.M.H., M.E.P., and F.J.D. performed data analysis and wrote the manuscript.

## Funding

This work was supported by grants T32GM108563 and HD074831 from the National Institutes of Health.

## Conflicts of Interest

The authors declare no conflicts of interest.

## Data Availability

The data that support the findings of this study are available from the corresponding author upon reasonable request.
